# Supplementary material for: Risk factors of bloodstream infection in erythroderma from atopic dermatitis, psoriasis, and drug reactions: a retrospective observational cohort study
Source: PeerJ. 2024 Jul 11;12:e17701. doi: 10.7717/peerj.17701 (PMC11246620; doi:10.7717/peerj.17701)
Supplement: Supplemental Information 2 [file peerj-12-17701-s002.docx]

Codebook

SEX（1=male，2= female）

Chilling(1=Yes,2=No)

Diabetes(1=Yes,2=No)

Hypertension(1=Yes,2=No)

Coronary Disease(1=Yes,2=No)

Kidney Disease(1=Yes,2=No)

Liver Disease(1=Yes,2=No)

Steroid history(1=Yes,2=No)

Skin bacteria(1=Yes,2=No)
