# Supplementary material for: Risk factors of bloodstream infection in erythroderma from atopic dermatitis, psoriasis, and drug reactions: a retrospective observational cohort study
Source: PeerJ. 2024 Jul 11;12:e17701. doi: 10.7717/peerj.17701 (PMC11246620; doi:10.7717/peerj.17701)
Supplement: Supplemental Information 3 [file peerj-12-17701-s003.docx]

**Supplementary Table 1** The summary table of characteristics between BSI group and Non-BSI group.

|  | BSI (n=11) | Non-BSI (n=130) | P value |
| --- | --- | --- | --- |
| Male (%) | 10 (90.90%) | 108 (83.08%) | 0.69 |
| T (<36.1℃ or >38.5℃)(%) | 9 (81.82%) | 17 (13.82%) | <0.001 |
| Chilling (%) | 8 (72.73%) | 14 (10.77%) | <0.001 |
| Etiology of drug reactions (%) | 6 (54.55%) | 28 (21.54%) | 0.03 |
| Etiology of AD (%) | 4 (36.36%) | 53 (40.77%) | 0.81 |
| Etiology of psoriasis (%) | 1 (9.09%) | 49 (37.69%) | 0.09 |
| History of steroids (%) | 7 (63.64%) | 46 (35.38%) | 0.10 |
| Kidney disease (%) | 5 (45.45%) | 7 (5.38%) | 0.001 |
| Diabetes (%) | 4 (36.36%) | 20 (15.38%) | 0.09 |
| ALB (Median) | 28.00 g/L | 34.95 g/L | <0.001 |
| CRP (Median) | 39.00 mg/L | 14.73 mg/L | 0.02 |
| IL-6 (Median) | 77.49 pg/mL | 8.43 pg/mL | 0.29 |
| PCT (Median) | 0.48 ng/mL | 0.10 ng/mL | 0.006 |
| LDH (Median) | 286.0 U/L | 282 U/L | 0.66 |
| N/L ratio (Median) | 4.23 | 3.77 | 0.29 |
| ESR (Median) | 36.50 mm/h | 19.00 mm/h | 0.04 |
| WBC (Median) | 8.28 x10^9/L | 9.60 x10^9/L | 0.65 |

**Supplementary Table 2** The univariate logistic regression of BSI in erythroderma.

| Factors | B | S.E. | wals | OR | 95% CI | P value |
| --- | --- | --- | --- | --- | --- | --- |
| T (≤36.0°C or ≥38.5°C) | 3.33 | 0.82 | 16.37 | 28.06 | 5.58–141.14 | <0.001 |
| Chilling | 3.10 | 0.73 | 17.80 | 22.10 | 5.25–93.08 | <0.001 |
| Kidney disease | 2.68 | 0.72 | 13.92 | 14.64 | 3.57–59.99 | <0.001 |
| Etiology of drug reactions | 1.43 | 0.64 | 4.98 | 4.18 | 1.19–14.68 | 0.03 |
| Etiology of AD | -0.15 | 0.65 | 0.06 | 0.86 | 0.24–3.08 | 0.81 |
| Etiology of psoriasis | -1.80 | 1.06 | 2.86 | 0.17 | 0.02–1.33 | 0.09 |
| Sex (male) | 0.71 | 1.08 | 0.44 | 2.04 | 0.25–16.74 | 0.51 |
| History of steroids | 1.20 | 0.65 | 3.35 | 3.31 | 0.92–11.90 | 0.07 |
| Diabetes | 1.15 | 0.67 | 2.90 | 3.14 | 0.84–11.74 | 0.09 |
| Age (year) | 0.02 | 0.02 | 1.19 | 1.02 | 0.98–1.06 | 0.28 |
| ALB (g/L) | -0.16 | 0.06 | 7.75 | 0.86 | 0.77–0.96 | <0.01 |
| CRP (mg/L) | 0.01 | 0.01 | 5.73 | 1.01 | 1.00–1.02 | 0.02 |
| IL-6 (pg/mL) | 0.02 | 0.01 | 5.08 | 1.02 | 1.00–1.04 | 0.02 |
| PCT (ng/mL) | 0.07 | 0.03 | 4.57 | 1.07 | 1.01–1.15 | 0.03 |
| LDH (U/L) | -0.001 | 0.002 | 0.09 | 1.00 | 0.99–1.00 | 0.77 |
| N/L ratio | 0.07 | 0.04 | 3.00 | 1.08 | 0.99–1.17 | 0.08 |
| ESR (mm/h) | 0.03 | 0.01 | 3.68 | 1.03 | 1.00–1.06 | 0.06 |
| WBC (x10^9/L) | -0.04 | 0.08 | 0.22 | 0.96 | 0.82–1.13 | 0.64 |

**Supplementary Table 3** The Chi-square test to check the dependency between temperature and chilling. There was significant dependency of temperature and chilling with the p value < 0.05.

| Factors | Chilling | No chilling |
| --- | --- | --- |
| T (≤36.0°C or ≥38.5°C) | 8 | 18 |
| T (36.1°C - 38.4°C) | 14 | 94 |

**χ2 = 4.84，dƒ=1, p=0.03**

**Supplementary Table 4** The multivariate logistic regression of BSI in erythroderma.

| Factors | B | S.E. | wals | OR | 95% CI | P value |
| --- | --- | --- | --- | --- | --- | --- |
| T (≤36.0°C or ≥38.5°C) | 2.48 | 0.90 | 7.59 | 11.95 | 2.05–69.77 | 0.006 |
| Kidney disease | 1.43 | 1.34 | 1.14 | 4.19 | 0.30–58.23 | 0.29 |
| Etiology of drug reactions | -0.30 | 1.19 | 0.07 | 0.74 | 0.07–7.55 | 0.80 |
| ALB (g/L) | -0.09 | 0.08 | 1.35 | 0.91 | 0.78–1.06 | 0.25 |
| CRP (mg/L) | -0.003 | 0.008 | 0.16 | 1.00 | 0.99–1.02 | 0.69 |
| PCT (ng/mL) | 0.20 | 0.31 | 0.40 | 1.22 | 0.66–2.23 | 0.53 |
